# Testing Sexual Strategy Theory in Norway

**DOI:** 10.3390/bs14060438

**Published:** 2024-05-24

**Authors:** Mehmet Mehmetoglu, Ilmari Määttänen, Matthias Mittner

**Affiliations:** 1 Institute for Psychology, Norwegian University of Science and Technology, 7491 Trondheim, Norway; matthias.mittner@ntnu.no; 2 Department of Psychology and Logopedics, University of Helsinki, 00014 Helsinki, Finland; ilmari.maattanen@helsinki.fi; 3 Institute for Psychology, UiT—The Arctic University of Norway, 9019 Tromsø, Norway

**Keywords:** mate choice, mate selection criteria, sexual strategy theory, sexual selection, mating mind

## Abstract

Choosing a romantic partner for a long-term relationship is one of the most significant decisions one makes during our lifetime. We have inherited an evolved framework from our ancestors that contains traits, as well as preferences for these traits, to solve this task. We use this framework consciously or unconsciously to choose prospective romantic partners. Following this reasoning, sexual strategy theory (SST) has been proposed for predicting which traits women and men prefer in a romantic partner for a long-term relationship. These predictions were empirically tested in the current work based on a sample of 1193 Norwegian adolescents who responded to an online questionnaire. We implemented the study hypotheses, derived from SST, in three statistical models, which were tested using structural equation modeling. In brief, our results revealed that women only valued resources more than men when we controlled for materialistic traits. This finding contrasts with SST’s prediction that women would value resources more than men, independently of other variables. As for the second prediction that men value physical attractiveness more than women, this pattern existed universally and was independent of, for instance, how egalitarian they were. We thus conclude that SST was only partially supported and that variables that may reflect societal circumstances (e.g., wealth, gender, equality) should be considered when examining the mate choice behavior of women and men. The theoretical and practical implications of the study are also discussed.

## 1. Introduction

The decision to choose a partner for a long-term romantic relationship (i.e., *mate choice*) is claimed to be one of the most critical choices made by any individual. This decision will have substantial positive or negative consequences for almost every aspect (e.g., relationship satisfaction, number of offspring, etc.) of a human being’s life (see, for instance, Fletcher et al. [1]). However, the ambit of the effects of such a vital decision is not limited to the person alone. It carries implications for their descendants across many future generations. This issue has ostensibly been a central aspect of human life since the times when our hunter-gatherer ancestors wandered the plains of Pleistocene Africa [2].

It was clearly in the interest of ancestral men and women to be sensitive to and act on the cues that could lead to a mate with desired traits. Their preferences for specific traits in potential mates were shaped by the mechanisms of *sexual selection*. Darwin [3] suggested that such traits might evolve if they are sexually selected (i.e., increasing reproductive success). Human mate choice behavior has evolved to focus on and exploit the reproductive potential and investment made by the partners [4]. Reproductive potential includes the genetic or other resources an individual can potentially invest in children, whereas reproductive investment is the actual use of these resources to promote the well-being of children [4]. The combination of the above two terms defines the individual’s mate value [5], and individuals with a higher mate value are more highly desired by potential mates [6]. From that point of view, human mate preferences today are not a random or arbitrary phenomena that exist only temporarily and change constantly. Instead, they are, to a large degree, products of our ancestors’ mating mind.

By benefiting from a variety of perspectives (evolutionary, social exchange, etc.) and theories/hypotheses (*parental investment*, i.e., the sex that invests more in the offspring will be more choosy; *sexy son*, i.e., women tend to choose males with the genes to create attractive sons), evolutionary psychology scholars have empirically endeavored to identify mate choice/selection criteria, commencing seriously with Hill’s [7] work back in the middle of the twentieth century (see [8]). Hill [7] includes the following eighteen features in his original mate selection survey, which are worth mentioning here. These are dependable character, emotional stability and maturity, pleasing disposition, mutual attraction and love, good health, desire for home and children, refinement and neatness, good cook and housekeeper, ambition and industriousness, chastity, education and intelligence, sociability, similar religious background, good looks, similar educational background, favorable social status, similar political background, and good financial prospects.

Hill’s [7] instrument was indeed used as a basis for a number of studies [9,10,11,12,13] on mate selection up until the 2000s, also including some recent attempts [14,15,16]. Although Hill’s [7] items were subject to slight amendments in these studies, no substantial changes appear to have been made throughout these years. By identifying some of the drawbacks (e.g., including student samples, neglecting relationship status, etc.) of this instrument, Schwarz and Hassebrauck [8] developed their own scale including no less than 82 items, which they subsequently subjected to a factor analysis, yielding twelve factors based on a large sample of 21,245 German citizens within the age range of 18–65 that were a in long-term relationship.

In addition to the descriptive listing of mate choice criteria, what is scientifically more interesting is to investigate the evaluation of these criteria by men and women separately. Consequently, Buss [12] conducted a comprehensive cross-cultural study including more than ten thousand respondents from thirty-three societies including Zambia, China, Greece, Norway, USA, Australia, and Brazil. Buss [12] found clear evidence for the evolutionary psychological hypotheses, in that significant sex differences were found as far as mate preferences were concerned. In summary, on average, women valued financial prospects and ambition more than men, whereas men placed more emphasis on physical attractiveness compared to women. This finding implies that these two preferences may indeed have evolved through sexual selection. Buss [17] interpreted this finding by stating that human females may have evolved species-typical psychological mate preferences for mates who display cues indicating the availability of resources and skills for resource acquisition. Resources were salient in ensuring the well-being and safety of both the mother and offspring, as well as increasing the likelihood of the offspring reaching the reproductive age to pass on their genes to subsequent generations.

These studies appear to have laid the foundation for the development of the *sexual strategy theory* (SST) by Buss and Schmitt [18,19]. SST is built around two main dimensions, namely the temporal duration of mating (ranging from short-term casual sexual encounters to long-term committed mating relations) and biological sex (male, female) resulting in a variety of sexual strategies with the aim of increasing reproductive success. When it comes to long-term relationships, SST posits that women’s mating strategy will prioritize men who are both able to acquire resources and willing to invest those resources into the family. Conversely, the theory predicts that men would prioritize physical attractiveness, since appearance provides observable cues to youth and health, and hence fertility. These attributes are widely recognized for their role in increasing the chances of successful reproduction, resulting in offspring with robust immune systems, and perpetuating good genetic material to future generations.

Although Buss and Schmitt [19] suggested that there is abundant empirical evidence for supporting these two SST predictions, there is still a debate as to whether women’s preference for resources and men’s preference for attractiveness are actually evolved. This doubt applies in particular to the former, which is also apparent in Buss’s [17] reply to Smuts with the title “Do Women Have Evolved Mate Preference for Men with Resources”. The biologist Rosenthal, in his comprehensive book on mate choice [20], took an even more unconvinced stance and suggested that these predictions are too simplistic and neglect salient environmental effects (i.e., social, cultural and economic factors) completely.

There is indeed some empirical evidence that environmental factors influence mate preferences. For instance, Zentner and Mitura [21] found that gender equality reduces sex differences in mate preferences. Yet another study revealed cultural influences by identifying systematic differences between Eastern and Western respondents regarding their mate preferences Thomas et al. [22]. Interestingly, the heritability of differences in mate preferences is low [23], which may indicate cultural flexibility of preferences. Environmental influences and individual characteristics, such as self-perceived mortality salience, may influence mate preferences. In the case of high mortality salience for example, individuals may become less selective and compromise or deviate from their stricter mate requirements [24].

Mate preferences also seem to change in societies over time, even within the relatively short time span of years or a few decades. A study about women’s matrimonial ads in France found that mate preferences were relatively stable from the 1920s through the 1960s, when the critical importance of material needs started decreasing and the importance of personality criteria started increasing again [25]. A study by Lu et al. [26] highlighted the influence of economic situation on the mate preferences of females. For example, “good father” attributes (e.g., staying at home, caring, considerate) are emphasized in stable economic situations, whereas ”good provider” attributes (e.g., good income, ambitious, good education) are considered more important under poor economic conditions.

By observing these somewhat contrasting theories and findings, Hou et al. [27] recently invited scholars to study the role of environmental factors in the influence of resources on mate choice decisions, as they observed a decreasing importance of economic resources in their empirical work.

Plainly put, SST predicts that physical attractiveness will be more important to men whereas resources will be more salient for women, regardless of any environmental factors. To be able to test these two predictions in their entirety, we identified two powerful concepts that comprise different environmental factors. These are materialism [28] and egalitarianism [29]. Briefly, materialism refers to the importance people attach to worldly possessions [30], and when a large share of a society scores highly on this dimension, a materialistic culture can be said to exist [28]. Egalitarianism, in summary, is the belief in the idea and ideal of equality [31], and when a considerable portion in a society possess this belief, that society will be defined as egalitarian.

After considering the studies and reasoning presented thus far, we suggested the following hypotheses for investigation in the current study. By doing so, we aimed to answer the research questions and gain a deeper understanding of the complex and mysterious behavior of mate selection.

H1: Women value resources more than men

H2: Men value physical attractiveness more than women

H3: Women value resources more than men, even when keeping materialistic orientations equal for both sexes

H4: Men value physical attractiveness more than women, even when keeping egalitarian orientations equal for both sexes

Building on the reasoning behind the previously mentioned hypotheses that relate to patterns between sexes, we suggested two additional hypotheses to test. The outcomes of these tests will provide valuable insights into patterns within the sexes as well.

H5: More materialistic women value resources more strongly than less materialistic women

H6: More egalitarian men value physical attractiveness less strongly than less egalitarian men

**Figure 1:**
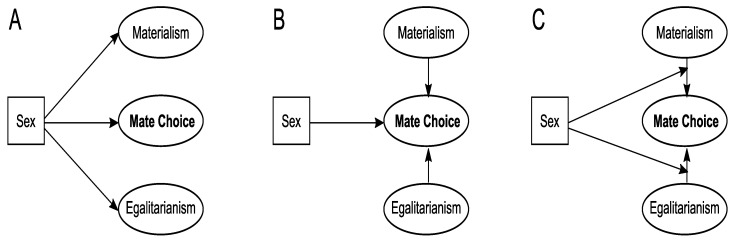
Overview of the current study’s models. (**A**) Model 1: Gender differences in mate choice criteria, materialism and egalitarianism (**B**) Model 2: Gender differences in mate choice criteria, corrected for materialism and egalitarianism (**C**) Model 3: Effect of materialism and egalitarianism on mate choice criteria moderated by gender.

The first two hypotheses (H1 and H2) are modeled in Figure 1A, the second two hypotheses (H3 and H4) in Figure 1B, and the last two hypotheses (H5 and H6) in Figure 1C. In addition to resources and physical attractiveness, we included several other mate choice criteria in these models which simultaneously provide more information about sex differences.

## 2. Methods

### 2.1. Sample

The necessary data to test the study’s hypothesized model were collected from a sample of 1193 Norwegian adolescents looking for a long-term partner or who were already in a relationship in the spring of 2021. An anonymous link to a web-based tool (https://nettskjema.no/, accessed on 19 March 2024) for collecting survey data was announced/shared with students at high schools and universities in two of the biggest cities in Norway. Testing the study’s model in a prototypical WEIRD (Western, educated, industrialized, rich, and democratic) society like Norway is in line with the the general consensus among evolutionary psychologists, e.g., [32], that studies of evolved human nature should be conducted in WEIRD societies. Subsequently, to estimate the model, we used full-information maximum likelihood (FIML, [33]) to account for missing data in the sample. Based on this sample, we observed that there were 109, 132, 151, 122, 144, 190, 146, 166, and 33 adolescents aged respectively 16, 17, 18, 19, 20, 21, 22, 23, and 24. In other words, the majority (67 percent) were university students, whereas the rest of the sample (about 33 percent) were high school students. There were 453 (about 39 percent) men in the sample, and the remaining 719 (about 61 percent) were women. Moreover, 636 (about 55 percent) of the sample were currently not in a romantic relation, whereas the remaining 528 (about 45 percent) were in a romantic relation.

### 2.2. Data Accessibility

All raw data and full R codes are available online in the project’s repository at the Open Science Framework (OSF; https://osf.io/ak76r/?view_only=dddfffa2649d495abc1e791e2a94f7ba, accessed on 19 March 2024).

### 2.3. Measures

In total, 36 items mapping mate choice criteria were included in the questionnaire. This shortened list of 36 items was obtained from Schwarz and Hassebrauck [8]. More specifically, the respondents were asked to indicate how important each criterion from a list of mate choice criteria (e.g., rich, witty, attractive, etc.) was for choosing their current partner (for those in a relationship) or a prospective partner (for those not in a relationship). These items used a 5-point scale ranging from 1 (not important at all) to 5 (very important). Moreover, the concepts of materialism and egalitarianism were measured using 5 items each. Here, the respondents were asked to reveal the extent to which they agreed with the statements (e.g., “I like to own things that impress people”, “Everyone should have an equal chance and say”) contained in the 10 items. These items also used a 5-point scale ranging from 1 (totally disagree) to 5 (totally agree). The items mapping materialism and egalitarianism were obtained from Richins and Dawson [28] and Katz and Hass [29], respectively. All the study items are provided in Table 1.

### 2.4. Data Analysis

All the analyses were performed using the open-source R software 4.4.0 [34], including the packages psych [35] and lavaan [36]. We first performed an exploratory factor analysis (EFA) on the 36 mate choice criteria, the output of which was submitted to a subsequent confirmatory factor analysis (CFA). In this circular analytical process between EFA and CFA, we decided to leave out 7 items, to be able to achieve convergent (i.e., low loadings removed) and discriminant validity (i.e., cross-loadings removed). As a result, the factor structure of the mate choice criteria included 29 items. We then added the 10 items representing materialism and egalitarianism to these 29 items and performed our final CFA, whose results are displayed in Table 1. All the CFA estimates were considered satisfactory, as the standardized loadings were above 0.5, the average variance extracted was close to or above 0.5, and the reliability coefficients were above 0.6. More importantly, the model fit to the data was clearly very good, as the RMSEA was below 0.05 and CFI was above 0.9 (see Table 1).

In line with the aim of the study, to be able to compare the two genders on the basis of regression coefficients later in the structural part of our model, we first needed to establish the metric invariance, meaning that the factor loadings were the same (i.e., items have the same meaning) for each gender. To do so, we performed a chi-square difference test between the equal form (configural) and equal loadings (metric) models. The results showed that the latter did not worsen the model fit (i.e., $\chi_{\mathrm{diff}}^{2}\left(27\right)=30.43,p=0.295$) providing evidence for measurement invariance as far as the loadings were concerned.

Subsequently, we proceeded to estimate the three structural models. The first model compared the two genders (i.e., means) based on the concepts of materialism, egalitarianism, and mate choice criteria (see Figure 1A). In a second step, we wished to investigate whether the gender effect on the mate choice critera would change when controlling for materialism and egalitarianism (see Figure 1B). We also estimated the effects of materialism and egalitarianism on mate choice criteria based on this model (see Figure 1B). Finally, having established metric invariance, we further estimated a third structural model, in which we examined the effect of the concepts of materialism and egalitarianism on mate choice criteria for each gender, as well as comparing the magnitudes of these effects (see Figure 1C). All three structural models demonstrated very good fit measures, with RMSEA values well below 0.05 and CFI values above 0.9 (see Table 2).

## 3. Results

### 3.1. Structural Model 1

**Figure 2:**
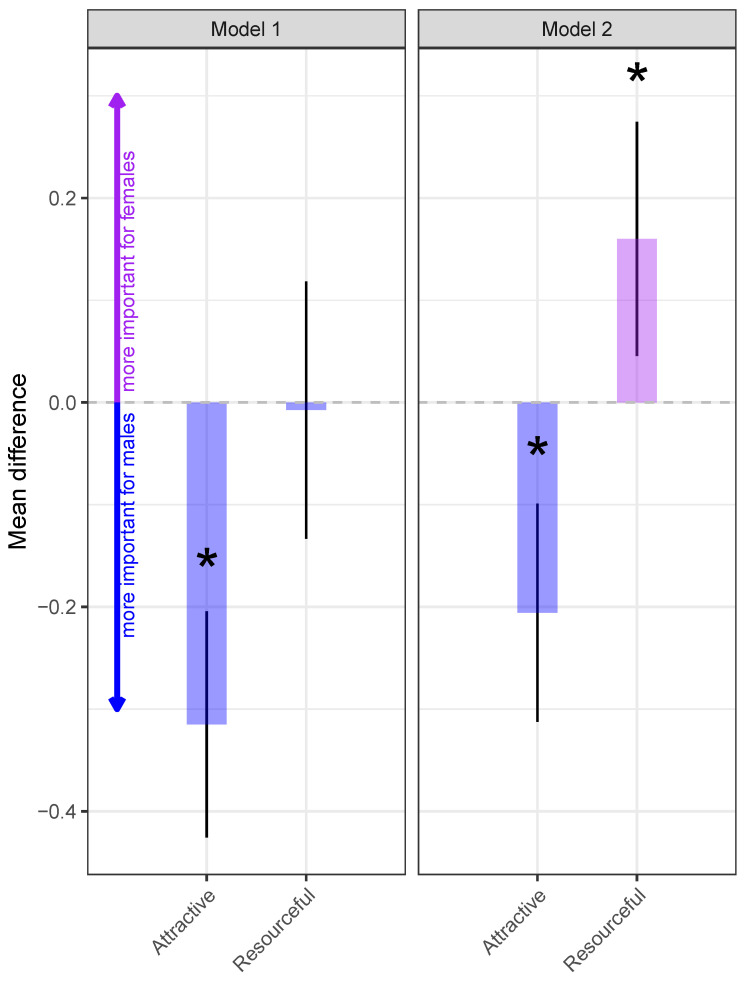
Comparison of men and women based on how important attractiveness and resourcefulness is for mate choice. Coefficients extracted from models 1 and 2 (see Table 3). * means statistical significance at alpha = 0.05.

The results concerning gender differences are visualized in figure Figure 2 (left) and detailed in Table 3. In line with our expectations (hypotheses derived from the SST), males emphasized attractiveness in prospective partners more than females (b=−0.32,z=−5.58,p<0.001). However, we failed to find support for the prediction that females would put a greater emphasis on resources compared to males (b=−0.01,z=−0.12,p=0.908). In addition, there were other differences between the two genders based on several of the latent variables included in our first structural model. More specifically, women scored on average higher than men on the mate choice criteria (i.e., preferring to a larger degree their partners to be) empathic (b=0.30,z=7.46,p<0.001), faithful (b=0.19,z=5.40,p<0.001), humorous (b=0.23,z=6.05,p<0.001), domestic (b=0.22,z=3.44,p<0.001), outgoing (b=0.19,z=3.29,p=0.001), and polite (b=0.22,z=5.24,p<0.001). Moreover, women were also on average more egalitarianism-orientated than men (b=0.25,z=6.14,p<0.001), while men scored higher on materialistic tendencies (b=−0.33,z=−5.26,p<0.001). These differences were statistically significant (see Table 3, Model 1).

There were no statistically significant differences between the two genders as far as the remaining two mate choice criteria, namely intelligence (b=−0.03,z=−0.92,p=0.357), and similarity (b=0.02,z=0.37,p=0.712) were concerned.

### 3.2. Structural Model 2

The unexpected null-finding regarding a greater resource-orientation for females in model 1 was anticipated by Rosenthal [20], who stated that environmental factors have to be taken into account when considering gender differences in mate choice preferences. Therefore, in the second model, we estimated the gender differences for the mate choice criteria, while controlling for materialistic and egalitarian tendencies (Figure 1B). Strikingly, the newly estimated model provided evidence for Rosenthal’s [20] hypothesis, as women scored higher on resource-orientation (as a mate choice criterion) compared to men when controlling for materialism and egalitarianism (b=0.16,z=2.74,p=0.006). On the other hand, the remaining pattern of gender differences remained unchanged, i.e., there was a stronger preference for attractiveness for males (b=−0.21,z=−3.78,p<0.001), while females scored more highly on empathic (b=0.22,z=5.73,p<0.001), faithful (b=0.14,z=4.02,p<0.001), humorous (b=0.21,z=5.58,p<0.001), domestic (b=0.27,z=4.19,p<0.001), outgoing (b=0.20,z=3.53,p<0.001), and polite (b=0.20,z=4.81,p<0.001). These findings are summarized in Figure 2 and Table 3.

As a consequence of these findings, the SST can be considered to be partially supported. However, with the caveat that environmental factors have a significant impact on how these tendencies evolve through sexual selection in modern society.

**Figure 3:**
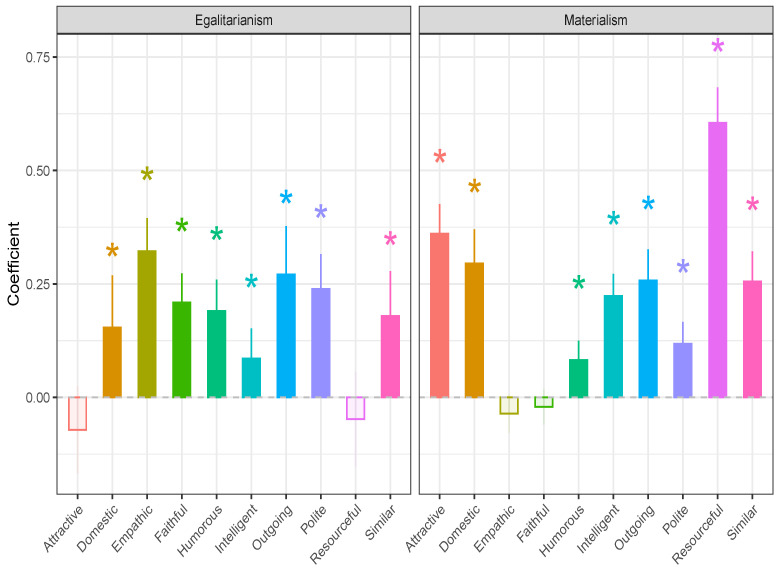
Effects of materialism and egalitarianism on mate choice criteria (extracted from structural model 2). Note: Empty bars reflect non-significant estimates. * means statistical significance at alpha = 0.05.

To further explore what effect environmental variables may have on the selection of prospective partners, we investigated the effect of materialistic and egalitarian tendencies on mate choice criteria directly (Figure 1B). According to Rosenthal’s [20] perspective, environmental factors affect mate choice. We can observe in Figure 3 (and Table 4) that the more egalitarian a person is, the more important are the traits domestic (b=0.15,z=2.62,p=0.009), emphatic (b=0.32,z=8.58,p<0.001), faithful (b=0.21,z=6.37,p<0.001), humorous (b=0.19,z=5.37,p<0.001), intelligent (b=0.09,z=2.51,p=0.012), outgoing (b=0.27,z=4.97,p<0.001), polite (b=0.24,z=6.05,p<0.001), and similar (b=0.18,z=3.54,p<0.001). Interestingly, egalitarianism does not have an effect on attractiveness (b=−0.07,z=−1.46,p=0.145) and resourcefulness (b=−0.05,z=−0.90,p=0.366).

Looking at the right-hand side of Figure 3, we next observe that the more materialistic a person is, the more important are the following mate choice criteria for her/him. These are attractive (b=0.36,z=10.83,p<0.001), domestic (b=0.29,z=7.65,p<0.001), humorous (b=0.08,z=3.82,p<0.001), intelligent (b=0.22,z=8.96,p<0.001), outgoing (b=0.26,z=7.34,p<0.001), polite (b=0.12,z=4.76,p<0.001), resourceful (b=0.60,z=15.20,p<0.001), and similar (b=0.26,z=7.55,p<0.001). The same figure shows also that materialism is not related to the two traits empathic (b=−0.04,z=−1.67,p=0.095) and faithful (b=−0.02,z=−1.05,p=0.293). The relationships between materialism and mate choice criteria included in our second structural model were all statistically significant (see Table 4). It is, however, worth emphasizing that materialism appears to be most strongly related to resourcefulness and attractiveness as mate-choice criteria.

### 3.3. Structural Model 3

**Figure 4:**
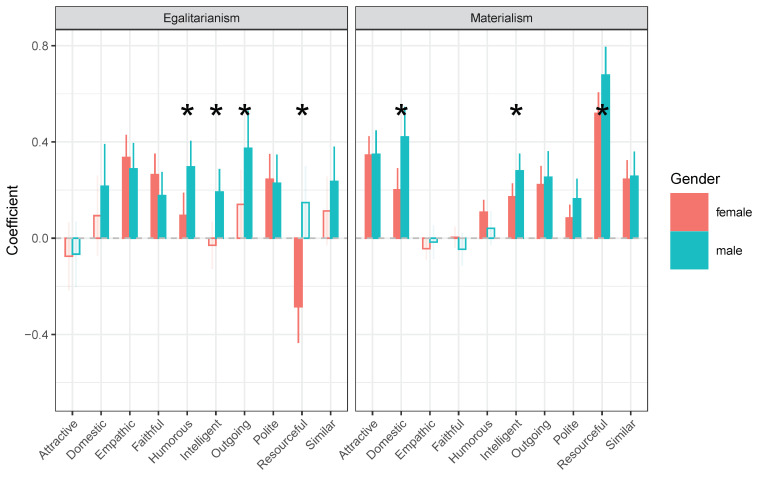
Comparison of men and women based on effects of materialism and egalitarianism on mate choice criteria (structural model 3). Note: Empty bars reflect non-significant estimates. * means statistical significance at alpha = 0.05.

Previously, in structural model 2, we identified the general pattern of how materialism and egalitarianism were associated with mate choice criteria for the whole sample, including both men and women. In structural model 3, we examined whether some of these associations might be different (i.e., stronger or weaker) for the two genders. As figure Figure 4 shows, the general patterns discovered in structural model 2 did indeed still apply to the whole sample as far as most of the traits were concerned.

Nonetheless, there appeared to be differences between men and women when it comes to the effects of materialism or egalitarianism on the mate-choice criteria domestic, humorous, intelligent, outgoing, and resourceful (see Table 5). The most striking effect in Figure 4 is that we observed that the effect of egalitarianism on resourcefulness was larger and negative for women (b=−0.29,z=−3.71,p<0.001). Previously (model 2), we discovered that egalitarianism was unrelated to resourcefulness as a mate choice criterion when combining men and women. The current finding suggests that, egalitarianism is predictive of preference for resourcefulness for females, where more egalitarian women will care less about resourcefulness as compared to less egalitarian women. On the other hand, the effect of egalitarianism for men on resourcefulness is positive though not significant (b=0.15,z=1.90,p=0.057). This difference between males and females was significant (Δb=−0.43,z=−3.95,p<0.001).

Our earlier findings from model 2 revealed that, although the more egalitarian, the more important the trait humorous, this relationship proved to be stronger for men (*b* = 0.30, *z* = 5.36, *p* < 0.001) than for women (b=0.10,z=1.99,p=0.047). That is, more egalitarian men were more preoccupied with humor than less egalitarian men (and that effect was reduced for women). For the trait of intelligence, the effect was positive for men (*b* = 0.19, *z* = 3.91, *p* < 0.001), i.e., that more egalitarian men valued more intelligent partners more than less egalitarian men. In contrast, for women, there was no such effect of egalitarianism on how much they valued intelligence in a prospective partner (*b* = −0.03, *z* = −0.60, *p* = 0.551). We saw a similar pattern regarding the trait of outgoingness (men: *b* = 0.37, *z* = 4.70, *p* < 0.001, women: (b=0.14,z=1.92,p=0.054)).

Finally, we discovered earlier that the more materialistic a person is, the more important the traits of being domestic, intelligence, and resourcefulness are for mate choice. Our results from model 3 further revealed that, while more materialistic men are more concerned about these features compared to less materialistic men, these effects were reduced for these traits for women (see Table 5).

## 4. Discussion and Conclusions

Sexual strategy theory predicts that women value resources more than men when considering partners for a long-term relationship. The current study did not find supporting evidence for this prediction in its broadest sense, as women and men valued resources similarly when averaging over the whole sample (H1 not supported). However, when we controlled for how materialistically oriented the respondents were, we identified a difference between the two sexes, i.e., women put more emphasis on resources than men (H3 supported). These findings suggest that systematic differences in the materialistic orientation between the genders hide the resource-preference effect for females. These differences may result from living in an advanced welfare state like Norway, providing women with a strong social and financial safety net that can reduce the importance of resourcefulness in prospective partners (see also [37]). Only when men and women were matched in terms of their materialistic mindset did the stronger preference for resourceful mates in females emerge.

We can conclude that, although resources may be an evolved mate preference for human females at an aggregate level, it is, however, not a static preference at the individual level, as women can diverge from this pattern when the socio-cultural conditions allow. This finding does, however, not undermine or reject a less literal reading of the SST. It just provides evidence for the necessity of incorporating contextual, social, and cultural factors into the consideration when investigating mate choice behavior. This nuance is also clearly reflected in Buss’s [17] reply to Smuts, which reads “As I pointed out several years ago, of course behavior depends on context. I have never asserted that female mate preferences are ‘obligate’ in the sense of invariably manifest in behavior regardless of context, and indeed have specified what some of these likely contexts are—the existence of resources that can be accrued and defended, the presence of men who vary in the resources they can offer, and the presence of men who are sometimes willing to channel those resource to a women and her children”. Despite the current finding, we should also stress the fact that, in a vast majority of cultures, women do still value resources more than men [17].

Sexual strategy theory predicts further that men value physical attractiveness more than women when considering a long-term relationship. The current study did indeed find evidence to support this prediction, in that we discovered that men in our sample valued physical attractiveness more strongly than women (H2 supported). Furthermore, even when we controlled for how egalitarian-orientated the respondents were, this pattern persisted (H4 supported). This finding points to how uncompromisingly important physical attractiveness is for mate-choice in men, a result that has been shown in numerous studies before [8,12,14,37,38,39]: Men, as compared to women, put more weight on physical attractiveness regardless of condition, culture, time, or context. In addition, men were more strongly concerned about physical attractiveness out of the ten traits that we included in the current work. It follows that this trait may indeed be hard-wired into the brains of human males.

We have so far established the fact that women appear to put less emphasis on resourcefulness as a desired trait in a non-materialistic setting like Norway. What alternative traits will women look for in their partner for a long-term relationship? Based on our results, these traits are emphatic, humorous, domestic, polite, faithful, and outgoing. Interestingly, this pattern stayed the same, even after controlling for the materialism and egalitarian orientation of the respondents.

These traits preferred by females all have in common that they refer to personal characteristics of the prospective mate, rather than their appearance or economic value. This finding aligns with previous literature suggesting that women value non-physical traits for long-term relations, while appearance is just as important for short-term relationships [37].

For the sake of discussion, we can qualitatively post-categorize these traits as emphatic/polite, humorous/outgoing, and domestic/faithful. Out of these traits, we suspect that the domestic/faithful trait is the one that substitutes the resourcefulness trait as far as women are concerned. In evolutionary terms, it will be difficult for your son/daughter to find a desired mate if she/he is not family-orientated, i.e., not doing housework and/or being unfaithful in the Norwegian or a similar context. There have even been studies [40] from Germany, for instance, suggesting that a man doing housework might lead to higher sexual frequency in marriage!

A potential explanation for why the traditional notion of resourcefulness may have been supplanted by the traits of domesticity and faithfulness among women could stem from the evolving socio-economic landscape in Norway. In contemporary Norwegian society, women are less dependent on men for financial stability, due to the robust social safety nets provided by the state and equal opportunities for employment [37]. Consequently, their reliance on men’s resourcefulness has diminished, prompting a broader search for resources beyond mere financial support. This shift is where the importance of domesticity and faithfulness emerges. Women now expect men to contribute to family well-being in various non-financial ways, such as actively participating in childcare, household chores, and nurturing the emotional fabric of the family. This expectation has become ingrained in younger generations, reshaping their understanding of partnership dynamics. Men, no longer burdened with the sole responsibility for financial provision, are increasingly developing skills associated with effective parenting and household management [41]. In essence, as women alleviate some of the financial pressures traditionally placed on men, they naturally desire an equitable distribution of domestic responsibilities. This mutual evolution has led to a more balanced and enriched environment for raising children, both quantitatively and qualitatively. Consequently, we can infer that this symbiotic exchange fosters the betterment of off-spring and strengthens familial bonds in the Norwegian setting, as well as in similar socio-economic contexts.

Moreover, this study ascertained that Norwegian men are more materialistic than women. Relatedly, the analyses further revealed that materialism orientation affects resource-seeking for women, i.e., more materialism leads to more resource-seeking. In other words, more-materialistic women value resources more than less-materialistic women (H5 supported). Extrapolating from our data, this finding may suggest that in a highly materialistic setting or culture, the chances can become even higher of an average woman seeking resources from a prospective partner. This finding generalizes (to an even more significant degree) to men in this sample. Another mate choice criterion that materialistic orientation influences is physical attractiveness, i.e., more materialism is associated with a stronger focus on physical attractiveness. This pattern applies equally to both men and women. That is, more materialistic men/women value physical attractiveness more than their less materialistic counterparts. The fact that materialistic people, in general, value both resources and physical attractiveness should not be surprising, as they want the best and most for themselves, a selfish approach which would serve the gene’s purposes (survival and reproduction) well. To put this popularly, genes will love materialistic humans!

The study’s findings show further that women are more egalitarian than men. However, we did not find any significant relationship between egalitarian orientation and the importance of physical attractiveness for mate-choice (H6 not supported). In other words, however egalitarian a person is, this does not influence that person’s interest in physical attractiveness as a mate criterion. This finding applies to both sexes, as the interaction was not significant. Nevertheless, it is worth noting that the non-significant relationship was in the negative direction (and stronger for women), as we would expect. We identified another interesting, significant relationship between egalitarian orientation and resource-seeking for women (but not for men). That is, more egalitarian women valued resources less than less egalitarian women. Previously, we spotted that more materialistic women valued resources more. In this respect, egalitarianism is competing with materialism as far as women are concerned. While both constructs, materialism and egalitarianism, are products of a complex interplay between genetic, cultural, and social factors, egalitarianism is shaped by culture [42] to a larger degree, while materialism has a stronger genetic predisposition [43]. Nevertheless, both constructs are situated in a broader socio-cultural context that modulates individual beliefs and behaviors.

More elaborately, the concept of “selfish genes”, which prioritize maximizing utility to enhance genetic propagation, manifests in various human behaviors, particularly in the relentless pursuit of material possessions such as money, cars, and houses (i.e., materialism). Conversely, some cultures have developed a counterpoint to this genetic drive in the form of egalitarianism. Unlike the innate impulses attributed to selfish genes, egalitarianism emerges from cultural evolution and promotes values that often oppose materialistic tendencies. Exaggerated for effect, in a hypothetical scenario, a materialistic individual might discriminate against another person when hiring, to secure personal benefits, exemplifying the influence of selfish genes. On the other hand, someone with egalitarian beliefs might actively resist such self-serving impulses, opting to treat all candidates fairly, even at a personal cost. This distinction highlights the tension between innate genetic drives and culturally developed values.

It is unsurprising that mate preferences, as shown through this work, have partially changed due to differing environmental demands in Norway. Environmental influences can be strong: gender equality, income inequality, wealth of the nation, and sex distribution may all influence the sex differences in mate preferences among humans. Sex differences within a country may also depend on the socioeconomic status of the individuals [44]. Even the parasite load may influence choices [45], which suggests that mate preferences are not necessarily rational and may involve lower-level learning or other ways to respond to the environment. The preferences may be susceptible to change, even in shorter periods, including the influence of the ovulatory cycle and the use of substances [46].

The “gender equality paradox” [47] states that sex differences are typically more pronounced in more gender-egalitarian countries such as Norway. This phenomenon is explained by deeply rooted or intrinsic sex differences in preferences, which materialize more quickly in countries where economic constraints are more limited [48]. This pattern applied to the differences between the two sexes’ mate preferences for physical attractiveness, whereas it fails to do so regarding their mate preferences for resourcefulness. One reason for this outcome could be the fact that women’s preference for resources may not be as hard-wired as men’s preference for physical features. This result suggests that women’s resource preference is a highly adaptable criterion within an extensive mate choice menu that has evolved since ancestral times.

Both human [49] and animal [50] studies show females mimic others’ mate choices and preferences. Cultural shifts, like Western societies moving from material to non-material priorities [25], could hasten and strengthen these changes through imitation, potentially creating distinct between-population differences fueled by social learning [51].

As far as limitations are concerned, first, this study used a cross-sectional design, based upon which we cannot directly make causal inferences. However, we have still tried to increase the credibility of the proposed relationships through compelling theoretical arguments. A second limitation is that, despite a large sample size, the sample was a convenience sample, which certainly makes the generalizability of these findings to the entire Norwegian population less certain. However, our main conclusions relate to differences between the genders. Any bias caused by the sampling method would have to affect the mate-choice preferences differentially for men and women in order to affect our conclusions. While a bias on mate-choice preferences caused by overrepresentation of young, highly-educated respondents is possible, it seems unlikely that this would have affected the two genders differentially. These two limitations could be circumvented in future research endeavors. In addition, the negative (but non-significant) relationship between egalitarian orientation and physical attractiveness reported earlier deserves more investigation in future research. The non-significant association in the current work may have been due to the sample composition, cultural context, or even our statistical model not including all the relevant variables. These factors could be considered in future attempts to study the possible effects of egalitarian orientation on the importance given to physical attractiveness.

Another related suggestion for future research is to test the results of this study (i.e., the link between materialism and resource-seeking, and egalitarianism and appearance) in countries known to score highly on materialism and in countries scoring highly on egalitarianism. One final suggestion could be to test the hypotheses from the current work with a sample including older participants (i.e., >24 years), to extend the results of this study over and above the adolescents included in this study. A comparative study examining the differences between the two mentioned age groups (<24 versus >24) would help us ascertain other possible reasons for the current study’s findings (i.e., women’s preference for resources) regarding adolescents. It may be the case that the importance of financial resources in a potential mate may not become fully relevant for women until they are no longer dependent on their parents’ income. Another suggestion for future research would be to explore whether adolescents in egalitarian societies prefer partners with good parenting qualities, even if they have limited resources.

Yet another limitation of the current study could be related to our operationalizing of the concept of “status”. In our study, we adopted a broad conceptualization of “status” (i.e., our item read “has high status”), which is one of the three items making up the overarching mate choice criterion “resourceful”, based on which we compared men and women. As other factors than only wealth and economic possessions, such as physical attractiveness and intelligence, may equally be the driving force behind “status” (see [52]), we cannot be confident that all the respondents associated “status” with wealth and economic possessions, which indeed was the premise for our analyses in the current study. As such, we suggest that future research adopts a more nuanced approach to operationalizing the concept of “status” with its possible sub-dimensions. Another limitation of this study was that we needed to control for social desirability when measuring the traits of egalitarianism and materialism. Finally, we should also stress that we only collected data in one gender-egalitarian country (i.e., Norway). We should have collected data from several other gender-egalitarian countries as well. This limitation could also be considered in future research.

## Figures and Tables

**Table 1:** Psychometric properties of the measurement model (standardized loadings, communalities, and reliability coefficients).

Latent Variable	Indicator	Loading	AVE	RRC
RESOURCEFUL	Rich	0.86	0.683	0.864
	Has high status	0.843		
	Successful in career	0.774		
EMPATHIC	Considerate	0.664	0.472	0.725
	Empathic	0.653		
	Understanding	0.741		
HUMOROUS	Witty	0.577	0.606	0.801
	Funny	0.865		
	Humorous	0.858		
INTELLIGENT	Intelligent	0.549	0.546	0.796
	Highly educated	0.872		
	Literate	0.76		
DOMESTIC	Good at cooking	0.75	0.509	0.741
	Good at housework	0.836		
	Family person	0.515		
ATTRACTIVE	Good looks	0.851	0.658	0.855
	Sexy	0.837		
	Attractive	0.74		
POLITE	Has good manners	0.54	0.505	0.728
	Polite	0.795		
	Well-behaved	0.769		
FAITHFUL	Honest	0.731	0.528	0.766
	Faithful	0.776		
	Trustworthy	0.67		
OUTGOING	Outgoing	0.713	0.492	0.745
	Spontaneous	0.719		
	Initiator	0.672		
SIMILAR	Has similar interests	0.659	0.428	0.598
	Has similar opinions	0.648		
MATERIALISM	I look up to people who own expensive homes, cars, and clothes	0.745	0.443	0.798
	I appreciate some extra luxury in my life	0.619		
	I like to own things that impress people	0.687		
	I feel that people possessing more things are happier	0.688		
	My life would be better if I owned certain things I do not have	0.577		
EGALITARIANISM	One should be kind to all people	0.652	0.445	0.801
	One should always find ways to help others	0.679		
	One should be concerned about the well-being of others	0.674		
	One should appreciate everyone as they are because we are all human beings	0.716		
	Everyone should have an equal chance and an equal say	0.612		

Note: AVE (average variance extracted), RRC (Raykov’s reliability coefficient).

**Table 2:** Fit indices for the three structural models.

Model	$\chi^{2}$	df	*p*-Value	RMSEA	CFI	TLI	SRMR
Model 1	2026.9	663	<0.0001	0.042	0.91	0.90	0.051
Model 2	2088.9	665	<0.0001	0.042	0.91	0.89	0.054
Model 3	2671.7	1299	<0.0001	0.043	0.91	0.90	0.056

**Table 3:** Mean differences between men and women based on mate choice criteria, materialism, and egalitarianism for both structural models 1 and 2.

	Model 1			Model 2		
	*b*	SE	*p*-Value	*b*	SE	*p*-Value
RESOURCEFUL	−0.01	0.06	0.908	0.16	0.06	0.006
EMPATHIC	0.30	0.04	<0.001	0.22	0.04	<0.001
HUMOROUS	0.23	0.04	<0.001	0.21	0.04	<0.001
INTELLIGENT	−0.03	0.04	0.357	0.01	0.04	0.880
DOMESTIC	0.22	0.06	<0.001	0.27	0.06	<0.001
ATTRACTIVE	−0.32	0.06	<0.001	−0.21	0.05	<0.001
POLITE	0.22	0.04	<0.001	0.20	0.04	<0.001
FAITHFUL	0.19	0.03	<0.001	0.14	0.03	<0.001
OUTGOING	0.19	0.06	0.001	0.20	0.06	<0.001
SIMILAR	0.02	0.06	0.712	0.05	0.06	0.383
MATERIALISM	−0.33	0.06	<0.001			
EGALITARIANISM	0.25	0.04	<0.001			

**Table 4:** Slopes for materialism and egalitarianism for the mate choice criteria (extracted from model 2).

	Materialism			Egalitarianism		
	*b*	SE	*p*-Value	*b*	SE	*p*-Value
RESOURCEFUL	0.60	0.04	<0.001	−0.05	0.05	0.366
EMPATHIC	−0.04	0.02	0.095	0.32	0.04	<0.001
HUMOROUS	0.08	0.02	<0.001	0.19	0.04	<0.001
INTELLIGENT	0.22	0.02	<0.001	0.09	0.03	0.012
DOMESTIC	0.29	0.04	<0.001	0.15	0.06	0.009
ATTRACTIVE	0.36	0.03	<0.001	−0.07	0.05	0.145
POLITE	0.12	0.02	<0.001	0.24	0.04	<0.001
FAITHFUL	−0.02	0.02	0.293	0.21	0.03	<0.001
OUTGOING	0.26	0.04	<0.001	0.27	0.05	<0.001
SIMILAR	0.26	0.03	<0.001	0.18	0.05	<0.001

**Table 5:** Difference in slopes between materialism/egalitarianism and mate-choice criteria for men and women extracted from model 3.

	Materialism				Egalitarianism		
	**Slope Diff**	**SE**	***p*** **-Value**		**Slope Diff**	**SE**	***p*** **-Value**
RESOURCEFUL	−0.16	0.07	0.022		−0.43	0.11	<0.001
EMPATHIC	−0.03	0.04	0.530		0.05	0.07	0.494
HUMOROUS	0.07	0.04	0.134		−0.20	0.07	0.005
INTELLIGENT	−0.11	0.04	0.013		−0.22	0.07	0.002
DOMESTIC	−0.22	0.08	0.004		−0.12	0.12	0.317
ATTRACTIVE	−0.00	0.06	0.963		−0.01	0.10	0.931
POLITE	−0.08	0.05	0.110		0.02	0.08	0.830
FAITHFUL	0.05	0.04	0.231		0.09	0.07	0.183
OUTGOING	−0.03	0.07	0.640		−0.23	0.11	0.027
SIMILAR	−0.01	0.06	0.849		−0.12	0.10	0.234

## Data Availability

The data presented in this study are publicly available at the Open Science Framework https://osf.io/ak76r/ (accessed on 19 March 2024).
